# The Editor's Letter-Box

**Published:** 1914-06-06

**Authors:** Humphrey Hirst

**Affiliations:** Chaplain. "Welborne," 270 Wellsway, Bath


### THE EDITOR'S LETTER-BOX.

### The Retrograde Policy of the Bath Guardians.

*To the Editor of* The Hospital.

Sir,—In your last issue under the head of "Retrograde Policy of the Bath Guardians" you lend yourself to the perpetration of a cruel injustice to every responsible person connected with the hospital of this Union when you publish as a fact: "The workhouse infirmary was in such bad repute amongst the sick poor that they refused to enter it, saying they would rather die at home."

I have often, in my pastoral work, heard the "rather-die-at-home" sentiment expressed by the sick poor in London, Bristol, and other places, and probably, in common with these places, it has often been expressed in Bath simply because of the deep-rooted antipathy to the workhouse among the self-respecting poor. I, however, challenge the accuracy of the statement if its *raison d'être* is to be sought for in the deficiencies or inefficiencies of our hospital as your article insinuates. Patients have repeatedly expressed to me their regret that they had not come in before, saying "If I had only known what it was like I would not have stayed out so long." During my nine years as chaplain I have received many letters of appreciation and gratitude from patients for their experience in our hospital. Only last Friday I was visiting an aged widow who was a patient less than a year ago and she said to me, "If I should be ill again I shall come back into the hospital." As a member of our Bath City Mission Committee I am in frequent contact with our three city missionaries, whose time is spent among the poor, but I have never heard a word from them harmonising in the least degree with your statement. I have, however, never yet been in "the perfect hospital," and I offer no comment on the worthy aims of those who make that their ideal.—Yours truly,

Humphrey Hirst, Chaplain.
"Welborne," 270 Wellsway, Bath, June 2, 1914.

[We gladly publish these two letters, showing, as they do, a sense of responsibility for the care of the sick poor lively enough to feel outraged at damaging allegations; but it is to be noticed that neither of our correspondents challenges a single fact that we have put forward. The sentence for which so much indignation is responsible reads, not as quoted by both writers, but as follows:—"Worst of all, *it was said* that there was no provision for the sick children, who had to be placed in beds between the adults, where room could be found for them, and that the workhouse infirmary was in such bad repute amongst the sick poor that they had refused to enter it, saying they would rather die at home." The words we have italicised are omitted from the quotation by our correspondents. We were repeating what was said, in that sentence, and Dr. Fuller's criticism we described lower down as a grave indictment, which it certainly is. To his and Dr. Curd's criticism we added the comment that, in view of the facts and recommendations put forward by Dr. Fuller, as Local Government Board inspector, and Dr. Curd, one of the members of the Board of Guardians, "the matter is one which the inhabitants of Bath cannot allow to remain in its present state." If there is enough indignation to denounce the reports which we quoted, why is there not enough force behind it to carry out the Local Government Board inspector's recommendations for reform? As we noted, the question was shelved by 30 votes to 20 at the meeting which occasioned our paragraph.—Ed. The Hospital.]

